# Ultrasound appearance of the kidney among radiology department attendees of a tertiary centre in Malawi

**DOI:** 10.12688/wellcomeopenres.18455.2

**Published:** 2023-02-02

**Authors:** Laura Carey, Bright Tsidya, Bazwell Nkhalema, Sylvester Kaimba, Karen Chetcuti, Elizabeth Joekes, Benno Kreuels, Marc Henrion, Jamie Rylance

**Affiliations:** 1 Malawi-Liverpool Wellcome Trust, Blantyre, Malawi; 2 Clinical Sciences, Liverpool School of Tropical Medicine, Liverpool, UK; 3 Radiology Department, Queen Elizabeth Central Hospital, Blantyre, Malawi; 4 Malawi College of Health Sciences, Blantyre, Malawi; 5 Kamuzu University of Health Sciences, Blantyre, Malawi; 6 Worldwide Radiology, Liverpool, UK; 7 Department of Implementation Research, Bernhard Nocht Institute for Tropical Medicine, Hamburg, Germany; 8 Department of Tropical Medicine, University Medical Center Hamburg-Eppendorf, Hamburg, Germany

**Keywords:** Ultrasound, Imaging, Africa, Malawi, kidney size, HIV

## Abstract

**Background:** Diagnostic and therapeutic decisions in nephrology in low-resource settings are frequently based on ultrasound assessment of kidney size. An understanding of reference values is critical, particularly given the rise of non-communicable disease and the expanding availability of point-of-care ultrasound. However, there is a paucity of normative data from African populations. We determined estimates of kidney ultrasound measures, including kidney size based on age, sex, and HIV status, among apparently healthy outpatient attendees of Queen Elizabeth Central hospital radiology department, Blantyre, Malawi.

**Methods: **We performed a cross-sectional cohort study of 320 adults attending the radiology department between October 2021 and January 2022. Bilateral kidney ultrasound was performed on all participants using a portable Mindray DP-50 machine and a 5MHz convex probe. The sample was stratified by age, sex, and HIV status. Predictive linear modelling was used to construct reference ranges for kidney size estimating the central 95 percentiles of 252 healthy adults. Exclusion criteria for the healthy sample were known kidney disease, hypertension, diabetes, BMI > 35, heavy alcohol intake, smoking and ultrasonographic abnormalities.

**Results: **There were 162/320 (51%) male participants. The median age was 47 (interquartile range [IQR] 34-59). Among people living with HIV 134/138 (97%) were receiving antiretroviral therapy. Men had larger average kidney sizes: mean 9.68 cm (SD 0.80 cm), compared to 9.46 cm (SD 0.87 cm) in women ( *p *= 0.01). Average kidney sizes in people living with HIV were not significantly different from those who were HIV-negative, 9.73 cm (SD 0.93 cm) versus 9.58 cm (SD 0.93 cm) ( *p* = 0.63).

**Conclusions:** This is the first report of the apparently healthy kidney size in Malawi. Predicted kidney size ranges may be used for reference in the clinical assessment of kidney disease in Malawi.

## Introduction

Patients with kidney failure routinely undergo ultrasonography as part of their assessment. In Malawi when laboratory tests for kidney function are delayed or unavailable, therapeutic decisions are frequently made on ultrasound assessment. Kidney ultrasound is standard practice in Malawi as part of the assessment of acute kidney injury to identify chronic damage in the face of competition for dialysis beds. Patients with evidence of chronic kidney impairment are unlikely to be prioritized for kidney replacement therapy. Therefore, in Malawi, abnormal kidney size and appearance on ultrasound is often used as a surrogate marker for chronic kidney disease.

An understanding of reference kidney size values is critical, particularly given the rise of non-communicable disease and the expanding availability of point-of-care ultrasound in the region. A number of studies have reported reference values for kidney size in healthy adults measured by ultrasonography ^1– 4^. Data on ultrasound kidney size measurements for Africa, however, are scarce. Furthermore, there are established physiological differences between populations which underlie the need for population-based estimates for examining kidney size. For example, for the same height, the vital capacity and the forced expiratory volume in one second are about 14% smaller in adults of African lineage compared to Caucasian and Asian populations ^5^. The purpose of this study was to investigate the normal ultrasound measurements of the kidney among adults in Malawi.

## Methods

Queen Elizabeth Central Hospital (QECH) is a 1,300-bed government hospital providing free healthcare to Blantyre. QECH is the largest tertiary and teaching hospital in the country which manages severe trauma cases from the Southern and Eastern regions, and less severe cases from areas located near the hospital ^6^. Malawi is a low-income country in South-East Africa, with an estimated adult HIV prevalence of 9% ^7^.

At QECH, we performed a cross sectional study of adults (≥18 years) attending the radiology department for any imaging modality, mostly relating to accidents and injury. Patients were approached for recruitment, Monday-Friday, 0700-1700. Exclusion criteria were people lacking capacity to consent with no proxy consent available. Radiology department attendees for imaging after accidents were targeted as they are less likely to have pre-existing kidney pathologies compared to other groups within the hospital. The sample was stratified by age, sex, and HIV status. The sample was stratified by HIV status to enable a separate reference range for those living with HIV.

### Sampling and laboratory methods

Point-of-care HIV testing was done for those with unknown status or no recent negative test. Data on serum creatinine or estimated glomerular filtration rate were not available.

### Ultrasound

Bilateral kidney ultrasound was performed by departmental sonographers experienced in performing kidney ultrasound, using a portable Mindray DP-50 machine and a 5MHz convex probe. For each individual, we evaluated the left and right kidney size, presence of hydronephrosis, loss of corticomedullary differentiation, echogenicity, and any other significant abnormality (such as cysts or pyonephrosis).

The examination was performed with the patient supine and the longitudinal dimensions of the kidneys were visually estimated to represent the largest longitudinal section. Quality control was performed before and after initiation of the study by experts to assess adequate image quality. Prior to commencement, a series of test images were reviewed by two experts for quality of view, detection of abnormalities and accuracy of length measurement. Where there was disparity, feedback was given to sonographers and further training in image acquisition. On data completion, 10% images were randomly selected for external review with expert opinion taken as the ‘gold standard’ against which to benchmark the accuracy of the sonographers’ measurements. Images with insufficient quality as deemed by experts were rejected (n=4).

Because kidney length is related to body height, the relative kidney length was calculated using the kidney length: body height ratio (KBR) by dividing the absolute kidney length (millimetres) by the body height (centimetres) for each kidney ^3^.

### Exclusion

For normal size range estimates, to represent a ‘healthy’ population as closely as possible, participants were excluded after recruitment if they reported diabetes, current heavy smoking (> 20 cigarettes/day) or heavy alcohol intake (> 50 alcohol drinks/week), and if body mass index (BMI) > 35. Data did not contribute to normal range estimates where there were significant imaging abnormalities; hydronephrosis, suspected pyonephrosis, and loss of corticomedullary differentiation.

### Statistical analysis

Statistical analyses were performed using R version 4.0.2 ^8^. Summary statistics were calculated for the cohort, described using either median and interquartile range (IQR) or mean and standard deviation for continuous variables depending on data distribution, and proportions for categorical variables. Two-sample t-tests or non-parametric tests, depending on data distribution, were used to compare variables between groups.

To generate estimates of expected mean kidney size, predictive linear modelling was used to estimate the central 95 ^th^ percentile for mean kidney size based on age and sex. To quantify the uncertainty of the lower and upper limits of these ranges, we fitted the same linear model to 1,000 bootstrap samples of the healthy dataset. Bootstrapped 95% confidence intervals were then constructed around the upper and lower limits of the prediction interval. Finally, a linear model was used to generate model fits of kidney size across the age ranges according to both sex and HIV status.

### Sample size

The known mean kidney bipolar length in adult males in the USA is 12.40 cm with a standard deviation of 0.90 cm ^9^. We aimed to estimate the bipolar length in the Malawi population with a margin of error of 0.15 cm using the following formula, where *n* is the sample size, *z* ^2^_*a*/2_ = 1.96 (95% confidence level), *σ* ^2^ = 0.90 and *d* = 0.15 cm.


$$n=\frac{\left(z^{2}{\;}_{a/2}\;x\;\sigma^{2}\right)}{d^{2}}=\frac{\left(1.96^{2}\;x\;0.9^{2}\right)}{0.15^{2}}=138$$

The number needed was inflated to 160 to cover for 15% unusable data. To recruit 50:50 HIV positive to negative, the total sample size was 320. 

## Results

**Figure 1.**
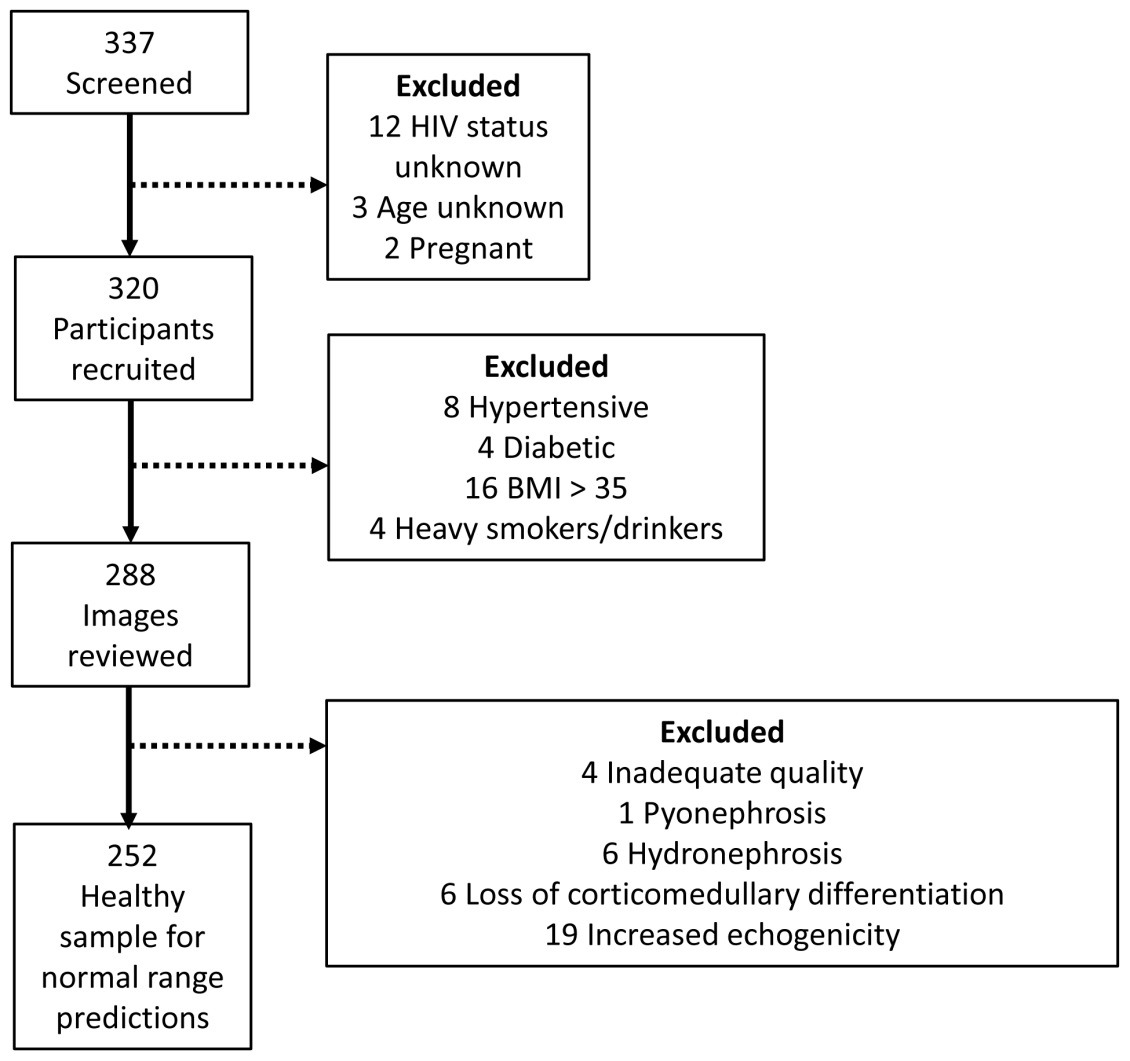
Study flow diagram demonstrating the number of participants recruited and selected for the healthy sample for normal range predictions.

Between 27 October 2021 and 31 January 2022, 320 participants were recruited. The study flow chart is summarised in Figure 1. Table 1 summarises the baseline characteristics of the participants. There were 162/320 (51%) male participants. The median age was 47 (interquartile range [IQR]34-59). Of those whose HIV status was positive, 138/320 (43%), 134/138 (97%) were receiving antiretroviral therapy. Tuberculosis (TB) history was known for 317/320, 303/320 (95%) had no prior TB history, 8/320 (3%) either received prior treatment or were receiving current treatment for TB, and 6/320 (2%) were diagnosed but never treated.

**Table 1.** Baseline characteristics of participants.

TABLE 1: Baseline characteristics of included participants			
	All (n = 320)	Female (n=158)	Male (n=162)
Age (years), median (IQR)	47 (34-59)	47 (36-59)	48 (33-57)
HIV positive, n (%)	138/320 (43%)	61/158 (39%)	77/162 (48%)
HIV status unknown, n (%)	12/320 (4%)	6/158 (4%)	6/162 (4%)
**Antiretroviral therapy**			
TDF/3TC/DTG, n (%)	125/138 (91%)	50/61 (82%)	75/77 (97%)
TDF/3TC/EFV, n (%)	6/138 (4%)	6/61 (10%)	0/77 (0%)
AZT/3TC/LPVr, n (%)	1/138 (1%)	0/61 (0%)	1/77 (1%)
ABC/3TC/DTG, n (%)	1/138 (1%)	0/61 (0%)	1/77 (1%)
AZT/3TC/ATVr, n (%)	1/138 (1%)	1/61 (2%)	0/77 (0%)
Not taking antiretroviral therapy, n (%)	4/138 (3%)	4/61 (7%)	0/77 (0%)
**Comorbidities**			
No TB history, n (%)	303/320 (95%)	149/158 (94%)	154/162 (95%)
Treated TB, n (%)	8/320 (3%)	4/158 (3%)	4/162 (2%)
Untreated TB, n (%)	6/320 (2%)	3/158 (2%)	3/162 (2%)
TB history not known, n (%)	3/320 (1%)	2/158 (1%)	1/162 (1%)
Hypertension, n (%)	8/320 (3%)	5/158 (3%)	3/162 (2%)
Hypertension unknown, n (%)	0/320 (0%)	0/158 (0%)	0/162 (0%)
Diabetes, n (%)	4/320 (1%)	2/158 (1%)	2/162 (1%)
Diabetes unknown, n (%)	19/320 (6%)	12/158 (8%)	7/162 (4%)
**Medications**			
No medication/unknown, n (%)	306/320 (96%)	151/158 (96%)	155/162 (96%)
NSAIDS, n (%)	10/320 (3%)	4/158 (3%)	6/162 (4%)
Thiazides, n (%)	3/320 (1%)	3/158 (2%)	0/162 (0%)
ACE inhibitors, n (%)	1/320 (0%)	0/158 (0%)	1/162 (1%)

TDF = tenofovir, 3TC = lamivudine, EFV = efavirenz, AZT = zidovudine, LPVr = lopinavir/ritonavir, ABC = abacavir, DTG = dolutegravir, ATVr = atazanavir/ritonavir, NSAIDS = nonsteroidal anti-inflammatories.

Social characteristics, symptoms, and reasons for attending the radiology department are shown in Table 2. Place of residence was known in 296/320, 218/320 (68%), reported living in an urban locality, 78/320 (24%) reported living rurally. proximity to the lake was known in 240/320, 14/320 (4%) reported living near Lake Malawi, where schistosomiasis is endemic.

**Table 2.** Table of social characteristics, symptoms, and reason for attending radiology department.

**Social**	
Current smoker, n (%)	15/320 (5%)
Smoking unknown, n (%)	23/320 (7%)
Drinks alcohol, n (%)	62/320 (19%)
Alcohol intake unknown, n (%)	22/320 (7%)
**Occupation**	
Professionals, n (%)	45/320 (14%)
Sales and services, n (%)	13/320 (4%)
Craft and related trades, n (%)	11/320 (3%)
Labourer, n (%)	7/320 (2%)
Service workers, n (%)	6/320 (2%)
Armed forces, n (%)	5/320 (2%)
Agriculture, n (%)	5/320 (2%)
Machine operators, n (%)	3/320 (1%)
Legislators/officials/managers, n (%)	2/320 (1%)
Other/no occupation, n (%)	223/320 (70%)
**Place of residence**	
Urban, n (%)	218/320 (68%)
Rural, n (%)	78/320 (24%)
Unknown, n (%)	24/320 (8%)
**Reason for attending**	
Fall, n (%)	98/320 (31%)
Road traffic accident, n (%)	59/320 (18%)
Assault, n (%)	22/320 (7%)
Bike injury, n (%)	5/320 (2%)
Collapsed structure, n (%)	3/320 (1%)
Burn, n (%)	1/320 (0%)
Other, n (%)	132/320 (41%)
**Symptoms**	
None, n (%)	287/320 (90%)
Abdomen/Lower back pain, n (%)	8/320 (3%)
Fever, n (%)	6/320 (2%)
Chest pain, n (%)	3/320 (1%)
Cough, n (%)	2/320 (1%)
Headache, n (%)	2/320 (1%)
Other, n (%)	9/320 (3%)
Unknown, n (%)	3/320 (1%)
**Imaging**	
X-ray, n (%)	192/320 (60%)
Ultrasound, n (%)	106/320 (33%)
Other, n (%)	22/320 (7%)

Physiology and ultrasound variables are provided in Table 3. The prevalence of hydronephrosis, increased echogenicity and loss of corticomedullary differentiation was low (2-6%). The mean size of the right kidney was 9.38 cm (SD 0.98 cm) in women, and 9.61 cm (SD 0.93 cm) in men. The mean size of the left kidney was 9.54 cm (SD 0.97 cm) in women and 9.76 cm (SD 0.90 cm) in men. Men had larger average kidney sizes: mean 9.68 cm (SD 0.80 cm), compared to 9.46 cm (SD 0.87 cm) in women ( *p* = 0.01). Average kidney sizes in HIV-positive participants were not significantly different from those who were HIV negative, 9.73 cm (SD 0.93 cm) versus 9.58 cm (SD 0.93 cm) ( *p* = 0.63).

**Table 3.** Physiology and ultrasound variables.

Variable	Overall	Female	Male
Height (cm)	162.40 (SD 99.01)	158 (153-163)	167 (160-172)
Weight (kg)	67.30 (IQR 57.00-76.67)	64 (56-80)	63 (59-73)
Body mass index (kg m ^-2^)	24.00 (IQR 22.00-28.00)	26 (23-31)	24 (21-26)
Systolic blood pressure (mm Hg)	136.00 (SD 23.61)	130 (116-158)	138 (125-156)
Diastolic blood pressure (mm Hg)	80.00 (SD 12.11)	80 (75-89)	81 (74-89)
Average kidney size (cm)	9.58 (SD 0.83)	9.46 (SD 0.87)	9.68 (SD 0.80)
Kidney size left (cm)	9.66 (SD 0.94)	9.54 (SD 0.97)	9.76 (SD 0.90)
Kidney size right (cm)	9.50 (SD 0.96)	9.38 (SD 0.98)	9.61 (SD 0.93)
Kidney length: height right (KBR)	0.59 (SD 0.06)	0.59 (SD 0.06)	0.58 (SD 0.06)
Kidney length: height left (KBR)	0.60 (SD 0.06)	0.60 (SD 0.06)	0.59 (SD 0.06)
Loss of corticomedullary differentiation, n (%)	6/320 (2%)	2/158 (1%)	4/162 (2%)
Increased echogenicity, n (%)	19/320 (6%)	9/158 (6%)	10/162 (6%)
Hydronephrosis, n (%)	6/320 (2%)	3/158 (2%)	3/162 (2%)
Pyonephrosis, n (%)	1/320 (0%)	1/158 (1%)	0/162 (0%)

**Figure 2.**
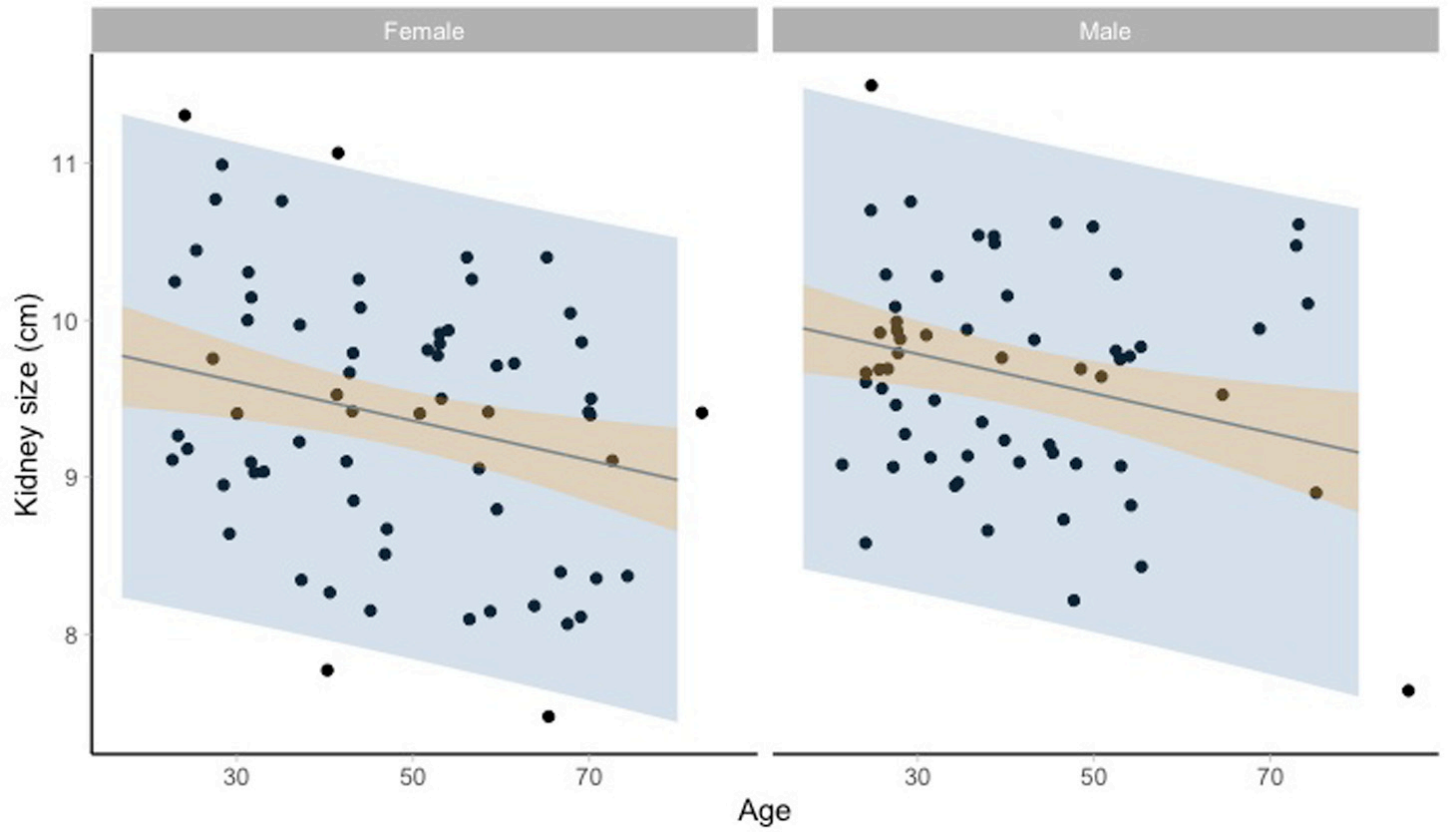
Kidney size range estimates dependent on age and sex for HIV negative participants. Model fit (grey line) of the linear regression model: lm(kidneysize ~ age + sex + hiv_status, data = noHIV) and 95% prediction intervals (blue) and 95% confidence intervals around the mean (orange).

**Figure 3.**
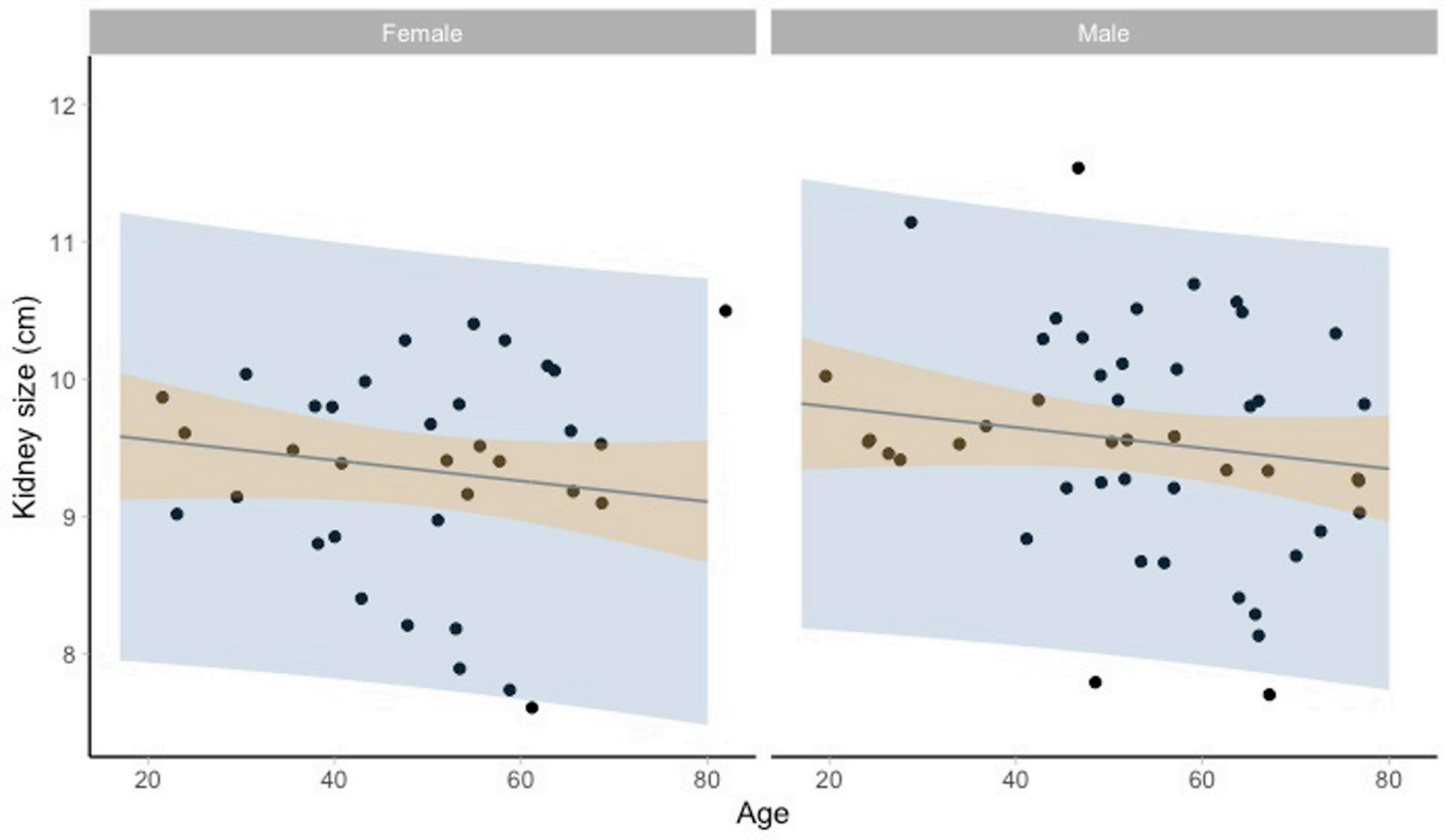
Kidney size range estimates dependent on age and sex for HIV positive participants. Model fit (grey line) of the linear regression model: lm(kidneysize ~ age + sex + hiv_status, data = HIV) and 95% prediction intervals (blue) and 95% confidence intervals around the mean (orange).

Absolute and relative kidney lengths are shown in Table 4. Absolute average (left and right) kidney lengths by sex and predicted range estimates with 95% confidence interval and bootstrapped upper and lower 95% prediction interval are shown in Table 5. The residuals plot for the multivariable model is in the GitHub repository ( *Extended data*) ^10^. Figure 2 and Figure 3 show the kidney size range estimates dependent on age, sex, and HIV status.

**Table 4.** Absolute and relative kidney lengths*. Abbreviation: KBR, ratio of kidney length in millimetres to subject height in centimetres. * Values are means (± 2 standard deviations).

	Age, years						
	18–29 *(n = 52)*	30–39 *(n = 55)*	40–49 *(n = 52)*	50–59 *(n = 58)*	60–69 *(n = 55)*	70+ *(n = 48)*	All *(n = 320)*
**Absolute kidney length, cm**							
Left	9.76 (8.19, 11.34)	9.77 (8.40, 11.13)	9.68 (7.73, 11.64)	9.55 (7.49, 11.62)	9.58 (7.43, 11.73)	9.56 (7.43, 11.68)	9.65 (7.76, 11.53)
Right	9.81 (8.15, 11.46)	9.36 (7.61, 11.10)	9.66 (7.66, 11.67)	9.38 (7.50, 11.26)	9.39 (7.38, 11.40)	9.41 (7.25, 11.57)	9.50 (7.57, 11.42)
**Relative kidney length, KBR**							
Left	0.60 (0.49, 0.70)	0.61 (0.52, 0.70)	0.59 (0.48, 0.71)	0.60 (0.46, 0.73)	0.59 (0.46, 0.72)	0.58 (0.45, 0.72)	0.60 (0.48, 0.71)
Right	0.60 (0.48, 0.71)	0.59 (0.46, 0.72)	0.59 (0.47, 0.71)	0.59 (0.47, 0.70)	0.58 (0.45, 0.71)	0.58 (0.44, 0.71)	0.59 (0.46, 0.71)
	Women Age, years						
	18–29 *(n =22)*	30–39 *(n =27)*	40–49 *(n =24)*	50–59 *(n =36)*	60–69 *(n =28)*	70+ *(n =21)*	All *(n =158)*
Absolute kidney length, cm							
Left	9.69 (8.80 -10.58)	9.71 (8.97-10.46)	9.56 (8.63-10.49)	9.59 (8.52-10.67)	9.27 (8.23-10.31)	9.42 (8.30-10.54)	9.54 (8.57-10.51)
Right	9.77 (8.92-10.62)	9.19 (8.20-10.18)	9.53 (8.42-10.64)	9.37 (8.52-10.22)	9.26 (8.34-10.17)	9.22 (8.02-10.42)	9.38 (8.40-10.36)
Relative kidney length, KBR							
Left	0.61 (0.55-0.67)	0.62 (0.57-0.67)	0.60 (0.55-0.66)	0.61 (0.54-0.67)	0.58 (0.52-0.64)	0.60 (0.53-0.66)	0.60 (0.54-0.66)
Right	0.62 (0.56-0.67)	0.59 (0.52-0.66)	0.60 (0.53-0.66)	0.59 (0.54-0.65)	0.58 (0.52-0.64)	0.58 (0.51-0.66)	0.59 (0.53-0.65)
	Men Age, years						
	18–29 *(n =30)*	30–39 *(n =28)*	40–49 *(n =28)*	50–59 *(n =22)*	60–69 *(n =27)*	70+ *(n =27)*	All *(n =162)*
Absolute kidney length, cm							
Left	9.82 (9.10-10.53)	9.82 (9.20-10.44)	9.79 (8.77-10.81)	9.49 (8.51-10.47)	9.90 (8.86-10.94)	9.66 (8.64-10.68)	9.76 (8.85-10.66)
Right	9.83 (9.01-10.66)	9.51 (8.78-10.24)	9.78 (8.87-10.68)	9.39 (8.29-10.48)	9.53 (8.44-10.62)	9.56 (8.58-10.53)	9.61 (8.68-10.55)
Relative kidney length, KBR							
Left	0.58 (0.54-0.63)	0.60 (0.56-0.65)	0.58 (0.52-0.64)	0.58 (0.51-0.65)	0.60 (0.53-0.66)	0.58 (0.51-0.64)	0.59 (0.53-0.65)
Right	0.59 (0.53-0.64)	0.59 (0.53-0.65)	0.58 (0.53-0.64)	0.57 (0.51-0.64)	0.58 (0.51-0.64)	0.57 (0.51-0.63)	0.58 (0.52-0.64)

**Table 5.** Absolute average (left and right) kidney lengths and predicted range estimates with 95% confidence interval and bootstrapped upper and lower 95% prediction interval. Absolute values are means (± 2 standard deviations) of the complete dataset n = 320. Predicted ranges and 95% confidence intervals were generated using a linear model to predict kidney size according to sex and age category: lm(kidneysize ~ age + sex, data= healthy). The model was then fitted to a bootstrapped sample of the healthy dataset with 1,000 replicates and bootstrapped 95% confidence intervals were constructed around the upper and lower limits of the prediction interval. Abbreviation: F, female, M, male.

	Age, years						
	18–29 *F = 22* *M = 30*	30–39 *F = 27* *M = 28*	40–49 *F = 24* *M = 22*	50–59 *F = 36* *M = 22*	60–69 *F = 28* *M = 27*	70+ *F = 21* *M = 27*	All *n = 320*
**Absolute average (left and right) kidney length, cm**							
Male	9.83 (8.60, 11.06)	9.67 (8.54, 10.79)	9.78 (8.16, 11.40)	9.44 (7.52, 11.35)	9.71 (7.82, 11.61)	9.61 (7.85, 11.36)	9.68 (8.09, 11.28)
Female	9.73 (8.18, 11.28)	9.45 (8.08, 10.82)	9.55 (7.76, 11.33)	9.48 (7.70, 11.27)	9.26 (7.48, 11.05)	9.32 (7.17, 11.47)	9.46 (7.72, 11.20)
**Predicted ranges (95% confidence interval)** *Healthy sample only (n=252)*							
Male	8.26-11.43 (9.60-10.14)	8.16-11.30 (9.53-9.96)	8.06-11.19 (9.43-9.82)	7.95-11.08 (9.30-9.71)	7.84-10.98 (9.16-9.63)	7.71-10.88 (8.98-9.56)	7.71-11.43 (8.98-10.14)
Female	8.05-11.22 (9.38-9.94)	7.95-11.09 (9.31-9.75)	7.84-10.98 (9.21-9.62)	7.74-10.87 (9.09-9.50)	7.63-10.77 (8.95-9.41)	7.50-10.67 (8.78-9.35)	7.50-11.22 (8.78-9.94)
**Bootstrapped lower and upper 95% prediction interval** *Healthy sample only (n = 252)*							
Male	Lower 8.08-8.46 Upper 11.15-11.67	Lower 8.00-8.35 Upper 11.07-11.49	Lower 7.88-8.27 Upper 10.99-11.35	Lower 7.74-8.20 Upper 10.88-11.25	Lower 7.59-8.14 Upper 10.76-11.16	Lower 7.41-8.06 Upper 10.63-11.10	Lower 7.41-8.06 Upper 11.15-11.67
Female	Lower 7.83-8.31 Upper 10.92-11.48	Lower 7.74-8.19 Upper 10.84-11.30	Lower 7.65-8.09 Upper 10.77-11.16	Lower 7.52-7.80 Upper 10.67-11.04	Lower 7.39-7.94 Upper 10.57-10.95	Lower 7.23-7.87 Upper 10.45-10.88	Lower 7.23-7.87 Upper 10.92-11.48

## Discussion

This is the first report of ultrasound appearances of normal kidneys in a Malawian population. In our cohort, kidneys were larger in males compared to females. Prevalence of ultrasound abnormalities such as hydronephrosis, increased echogenicity and loss of corticomedullary differentiation was low. Kidney size was not significantly different in people living with HIV versus those without HIV, meaning the table of predicted ranges can be applied to both groups. This may be related to the successful scale up and subsequent high antiretroviral therapy coverage in our cohort, 97% (134/138), compared to the subnational estimate of 92% for Blantyre ^11^.

We found kidney sizes in our Malawi cohort to be smaller than in a Nigerian population, which reported 10.20 cm (SD 0.81 cm) and 9.85 cm (SD 0.90 cm) for left and right kidneys ^12^. We also found kidney sizes in Malawi to be smaller than populations outside of SSA. For example, in the US the mean kidney bipolar length on ultrasound has been reported as 11.20 cm and 11.00 cm for left and right kidneys ^9^, in Kuwait 10.71 cm (SD 1.00 cm) cm and 10.68 cm (SD 1.40 cm) for left and right kidneys ^2^, and in Copenhagen, 11.20 cm and 10.90 cm for left and right kidneys ^1^.

These differences may be explained, in part, by population differences in height; however very few studies report relative kidney size, and none in Africa. After accounting for height using kidney length: body height ratio (KBR) our data suggest smaller relative kidney sizes among Malawians compared to European populations. For example, data from Croatia suggest KBRs in adults younger than 60 without kidney disease, are between 0.60 and 0.74 for the left kidney and 0.57 to 0.72 for the right kidney ^3^. In a Swiss autopsy series of 635 adults without diabetes or known kidney disease, mean (standard deviation) KBRs were 0.67 (0.07) for men and 0.69 (0.07) for women ^13^.

There were limitations to our study. We were unable to measure kidney function to confirm absence of pre-existing kidney disease. However, we excluded participants with comorbidities, social behaviours, and ultrasound abnormalities likely to affect glomerular filtration rate (GFR). The healthy sample may have included participants with kidney impairment. We relied on self-reporting of hypertension and diabetes and were unable to perform glucose measurements for diabetes screening. It is therefore possible that some participants with undiagnosed hypertension and diabetes contributed to the healthy sample. The quality control process did not include a formal assessment of interobserver variability. Only 10% of images were reviewed by experts for quality. There may be images remaining of insufficient quality which were not assessed. The relatively small sample for predicting kidney size within age and sex categories and sex, and the lower proportion of HIV-positive participants in the younger age categories may have biased the size estimates. We did not collect data on CD4 count or viral load, and future studies should aim to correlate kidney size with stage of HIV infection.

We recruited participants attending a tertiary centre for imaging following accidents as they were less likely to have a pre-existing kidney disease than other hospital-based cohorts. Future studies should aim to develop nomograms for adults and children, derived from a larger demographic sample. Ideally, these would also include GFR measurement, CD4 count and viral load for HIV positive participants.

In conclusion, we demonstrate the range of kidney sizes expected in adult Malawians without known kidney disease. We found a low prevalence of ultrasound abnormalities in our population. Our predicted size estimates within age categories can be referred to in the assessment of patients with kidney failure.

## Ethical statement

Participants gave written informed consent under ethical approvals from the College of Medicine Research Ethics Committee, University of Malawi (P.03/19/2625) and the Liverpool School of Tropical Medicine Ethics Committee (18-062). Study information including purposes, benefits and risk was provided to all participants in both English and Chichewa.

## Data Availability

Zenodo: careyla/Normal-kidney: v1.0.0, https://doi.org/10.5281/zenodo.7231616 ^10^ This project contains the following underlying data: baseline.csv210.0 kB fulldata.csv117.5 kB tidyclean2.csv117.6 kB uss_data.csv Zenodo: careyla/Normal-kidney: v1.0.0, https://doi.org/10.5281/zenodo.7231616 ^10^ This project contains the following extended data: Residualsplot Data are available under the terms of the Creative Commons Zero "No rights reserved" data waiver (CC0 1.0 Public domain dedication). Analysis code available from: https://github.com/careyla/Normal-kidney/tree/v1.0.0 Archived analysis code at time of publication: https://doi.org/10.5281/zenodo.7231616 ^10^ License: MIT Zenodo: STROBE checklist for ‘Ultrasound appearance of the kidney among radiology department attendees of a tertiary centre in Malawi’, https://doi.org/10.5281/zenodo.7231616 ^10^
