# Supplementary material for: Ultrasound appearance of the kidney among radiology department attendees of a tertiary centre in Malawi
Source: Wellcome Open Res. 2023 Feb 2;7:280. Originally published 2022 Nov 10. [Version 2] doi: 10.12688/wellcomeopenres.18455.2 (PMC9971658; doi:10.12688/wellcomeopenres.18455.2)
Supplement: Peer review history

# Peer review: Reviewer response for version 2

**DOI:** 10.21956/wellcomeopenres.20908.r54643

**Published:** 2023-02-16

**Authors:** Robert Kalyesubula

**Affiliations:** 1 Department of Medical Physiology, School of Biomedical Sciences, Makerere University, Kampala, Uganda; 2 Medical Research Council/UVRI & London School of Hygiene and Tropical Medicine Research Unit, Entebbe, Uganda

I have reviewed the responses and agree with indexing.

Is the work clearly and accurately presented and does it cite the current literature?

Yes

If applicable, is the statistical analysis and its interpretation appropriate?

Yes

Are all the source data underlying the results available to ensure full reproducibility?

Yes

Is the study design appropriate and is the work technically sound?

Yes

Are the conclusions drawn adequately supported by the results?

Yes

Are sufficient details of methods and analysis provided to allow replication by others?

Yes

Reviewer Expertise:

I am a nephrologist working in the area of determination of kidney function among people of African origin. I am also an actively practicing clinician from Uganda, Kampala.

I confirm that I have read this submission and believe that I have an appropriate level of expertise to confirm that it is of an acceptable scientific standard.

# Peer review: Reviewer response for version 2

**DOI:** 10.21956/wellcomeopenres.20908.r54644

**Published:** 2023-02-09

**Authors:** June Fabian

**Affiliations:** 1 Wits Donald Gordon Medical Centre, School of Clinical Medicine, Faculty of Health Sciences, University of the Witwatersrand, Johannesburg, South Africa

I am happy with the changes and happy to recommend the paper be accepted for indexing without further revisions.

Is the work clearly and accurately presented and does it cite the current literature?

Yes

If applicable, is the statistical analysis and its interpretation appropriate?

Yes

Are all the source data underlying the results available to ensure full reproducibility?

Yes

Is the study design appropriate and is the work technically sound?

Yes

Are the conclusions drawn adequately supported by the results?

Yes

Are sufficient details of methods and analysis provided to allow replication by others?

Partly

Reviewer Expertise:

Specialist physician and nephrologist, research interest is the epidemiology of kidney disease in African populations

I confirm that I have read this submission and believe that I have an appropriate level of expertise to confirm that it is of an acceptable scientific standard.

# Peer review: Reviewer response for version 1

**DOI:** 10.21956/wellcomeopenres.20466.r53281

**Published:** 2022-12-13

**Authors:** June Fabian

**Affiliations:** 1 Wits Donald Gordon Medical Centre, School of Clinical Medicine, Faculty of Health Sciences, University of the Witwatersrand, Johannesburg, South Africa

Thank you for this important work which, as the authors have stated, is much needed in African populations. 

 The authors state that in Malawi, because of the absence of additional diagnostic capabilities, kidney size on ultrasound is often used as a proxy for determining whether there is underlying chronic kidney disease in those who need dialysis (usually presenting as acute kidney injury). However, such interpretation is hampered by the absence of population-appropriate references for normal kidney size. They also make the point that POCUS is becoming more accessible and NCDs are rising in prevalence  - implying (perhaps) that kidney disease is on the rise and more clinicians will have access to POCUS. The authors screened 320 apparently healthy adults presenting at Queen Elizabeth Central hospital radiology department, Blantyre, Malawi. The authors found non-significant differences in kidney size between HIV + and HIV - participants, larger kidney sizes in men than women (expected), and relative kidney length seems to be smaller than in other studies (Nigeria, and high-income settings). They used predictive linear modelling to estimate kidney size stratified by age and sex that might be used in future. 

 I have a few questions that I would like to ask the authors for clarity: 

**1.** The stated purpose of the study was to investigate normal US measurements in apparently heathy Malawian adults. If so, I am not sure I understand why the sample was stratified by HIV status as we know that untreated HIV can result in loss of coticomedullary differentiation or larger than normal kidney sizes. It is a valuable finding in this study that for PLWHIV who have high ART coverage, kidney sizes are not significantly different from apparently healthy Malawians. Following on from this, it feels a little incongruent that NCDs are mentioned, yet excluded when we know that hyperfiltrating dabetics can have larger than normal kidney size and hypertension is linked to kidney size in the setting of CKD. One might argue that those with HIV should have been excluded?

 Perhaps asked another way, was the original study about kidney size in PLWHIV - and because no difference was found, these data could contribute to an apparently normal dataset. If so, it would be helpful to explain this in the paper and perhaps reframe it as such? Also, in clinical prcatice elsewhere, kidney size in the setting of HIV is/was used to ration access to short term dialysis support - perhaps this is also why the authors focused in HIV? If so, again, it would be very helpful to include this.

**2.** It would be helpful to describe the US methods in more detail that would enable replication: 

- Who did the ultrasound scans - was it only sonographers - any radiologists?
- How did you account for interobserver variability?
- Can you explain what QC measures were undertaken for each sonographer prior to starting the study? 

**3.** Tables 1, 3, 4 - since we describe differences in kidney size stratified by sex - can these tables describe the overall data and be stratified by sex with sample sizes for each category in the top tab for each column?

**4.** No screening was conducted (other than HIV) and it is well known that many with hypertension, kidney disease, and diabetes are unaware - so the apparently healthy population may indeed have had comorbidity. I think this is a significant limitation of the study and needs to be stated as such. Likewise, the relatively small sample sizes for predicting kidney size (by age caegory and sex) are also limitations that need to be stated.

 One nitpicky edit (if I may): sub-Saharan Africa (SSA): the "sub" should be capitalised ("Sub-Saharan") - and using this terminology has been questioned as colonial in origin - perhaps for the authors to consider that African / African populations might suffice?

Is the work clearly and accurately presented and does it cite the current literature?

Yes

If applicable, is the statistical analysis and its interpretation appropriate?

Yes

Are all the source data underlying the results available to ensure full reproducibility?

Yes

Is the study design appropriate and is the work technically sound?

Yes

Are the conclusions drawn adequately supported by the results?

Yes

Are sufficient details of methods and analysis provided to allow replication by others?

Partly

Reviewer Expertise:

Specialist physician and nephrologist, research interest is the epidemiology of kidney disease in African populations

I confirm that I have read this submission and believe that I have an appropriate level of expertise to confirm that it is of an acceptable scientific standard, however I have significant reservations, as outlined above.

# Response

**Published:** 2023-01-09

**Authors:** Laura Carey

**Affiliations:** University of Bristol, UK

1. Thanks for making this very important point. The original plan to stratify by HIV status was because we wanted sufficient power to detect a difference in kidney size, had there been one. With the high prevalence of HIV in Malawi, it was important for us to provide a reference range for both people living with HIV and those without, expecting there to be a difference. We agree with both statements. Untreated HIV can result in larger than normal kidney sizes. However, perhaps reassuringly, kidney sizes were not significantly different from apparently healthy Malawians in our study. Which could be related to high ART coverage, as you suggest. Given that we found little differences one could argue for combining the ranges. It is our experience in Blantyre, that kidney size regardless of HIV status is used to ration access to short term dialysis support. We have added the statement “ *The sample was stratified by HIV status to enable a separate reference range for those living with HIV” *to hopefully give more clarity to the rationale behind the stratification. With regards to NCDs, the reported prevalence is lower than HIV. The argument for exclusion is related to minimizing selection bias in the context of a hospital-based cohort without access to GFR measurement. We therefore excluded those who were more likely to have abnormal GFR (diabetics, and hypertensives).

 2. Sonographers performed the scans. Interobserver variability was not formally assessed, however, quality control was undertaken by two independent experts (including one radiologist). Prior to starting data collection, experts reviewed a series of test images performed by the sonographers and gave feedback and further training. After completion of data collection, 10% images were reviewed by the experts and images of insufficient quality rejected, accepting that there were likely to be images of insufficient quality remaining in the sample. This approach was chosen for pragmatic reasons, and is a limitation of the study which has now been made more explicit in the methods and limitations section. Methods: *Prior to commencement, a series of test images were reviewed by two experts for quality of view, detection of abnormalities and accuracy of length measurement. Where there was disparity, feedback was given to sonographers and further training in image acquisition. On data completion, 10% images were randomly selected for external review with expert opinion taken as the ‘gold standard’ against which to benchmark the accuracy of the sonographers’ measurements. Images with insufficient quality as deemed by experts were rejected (n=4).* Limitations: *The quality control process did not include a formal assessment of inter-observer variability. Only 10% of images were reviewed by experts for quality. There may be images remaining of insufficient quality which were not assessed. *  

 3 *. *Thank you for this helpful comment, the three tables are now presented stratified by sex as suggested.  

 4. We have now expanded the limitations section to makes these limitations clearer: *There were limitations to our study. We were unable to determine measured kidney function to confirm absence of pre-existing kidney disease. However, we excluded participants with comorbidities, social behaviours, and ultrasound abnormalities likely to affect glomerular filtration rate (GFR). The healthy sample may have included participants with kidney impairment. We relied on self-reporting of hypertension and diabetes and were unable to perform glucose measurements for diabetes screening. It is therefore possible that some participants with undiagnosed hypertension and diabetes contributed to the healthy sample. The quality control process did not include a formal assessment of inter-observer variability. Only 10% of images were reviewed by experts for quality. There may be images remaining of insufficient quality which were not assessed. The relatively small sample for predicting kidney size within age and sex categories and sex, and the lower proportion of HIV-positive participants in the younger age categories may have biased the size estimates.*

# Peer review: Reviewer response for version 1

**DOI:** 10.21956/wellcomeopenres.20466.r53279

**Published:** 2022-11-25

**Authors:** Robert Kalyesubula

**Affiliations:** 1 Department of Medical Physiology, School of Biomedical Sciences, Makerere University, Kampala, Uganda; 2 Medical Research Council/UVRI & London School of Hygiene and Tropical Medicine Research Unit, Entebbe, Uganda

This is a wonderful study undertaken by Carey *et al.* in an attempt to report on the normal kidney sizes among people from Malawi. The researchers provide very useful information that would help in determination of kidney pathology in SSA.

 I have a few comments that may need to be addressed:

1. The introductory statement sounds rather interesting. I would argue that most low-resource centers do not have access to ultrasound scans and therefore it is unlikely that decisions to treat kidney disease would be based on an ultrasound scan - perhaps proteinuria which is available on a dipstick. You may want to have this statement restricted to Malawi.
2. How was selection bias minimized in the selection of the participants? People who present to hospitals are sick and are therefore less likely to represent the general population. 
3. The use of creatinine or proteinuria would have been of additional value in excluding those with underlying kidney dysfunction at least GFR less than 60mls/min/1.73m2. Why was this not done?
4. Please provide more details on how you did the radiography. Was the interpretation done centrally or was this as used in routine practice? What happened if there was a disagreement in the 10% of images which were randomly selected for routine control?
  In the exclusion criteria; was diabetes only on self report? How reliable is this in excluding DM? I believe this would be a great confounder in the size of the kidney as would HIV since the kidneys tend to be bigger. Many DM patients do not know that they have it.
5. The standardization for body size is a strong point for this study. Is there any major difference in size between the rural and urban dwellers in the study and overall in Malawi?

The STROBE guidelines for cross sectional studies were dully followed which streamlined the reporting.

Is the work clearly and accurately presented and does it cite the current literature?

Yes

If applicable, is the statistical analysis and its interpretation appropriate?

Yes

Are all the source data underlying the results available to ensure full reproducibility?

Yes

Is the study design appropriate and is the work technically sound?

Yes

Are the conclusions drawn adequately supported by the results?

Yes

Are sufficient details of methods and analysis provided to allow replication by others?

Yes

Reviewer Expertise:

I am a nephrologist working in the area of determination of kidney function among people of African origin. I am also an actively practicing clinician from Uganda, Kampala.

I confirm that I have read this submission and believe that I have an appropriate level of expertise to confirm that it is of an acceptable scientific standard, however I have significant reservations, as outlined above.

# Response

**Published:** 2023-01-09

**Authors:** Laura Carey

**Affiliations:** University of Bristol, UK

"The introductory statement sounds rather interesting. I would argue that most low-resource centers do not have access to ultrasound scans and therefore it is unlikely that decisions to treat kidney disease would be based on an ultrasound scan - perhaps proteinuria which is available on a dipstick. You may want to have this statement restricted to Malawi."  

**Response: **This is a very good point, thank you for raising it. The introduction has been changed to only refer to practices in Malawi.  

 "How was selection bias minimized in the selection of the participants? People who present to hospitals are sick and are therefore less likely to represent the general population."   

**Response:** Thank you for raising this important question. We employed several methods to minimize selection bias in the population presenting to hospital. Firstly, because we wanted to ensure participants would be unlikely to have pre-existing kidney disease, we selected those who were attending for imaging and injury as a result of accidents. Secondly, we excluded participants who were pregnant, diabetic, hypertensive or smokers and alcohol drinkers. Thirdly, we excluded those who had imaging abnormalities identified on ultrasound.  

 "The use of creatinine or proteinuria would have been of additional value in excluding those with underlying kidney dysfunction at least GFR less than 60mls/min/1.73m2. Why was this not done?"  

**Response:** We are wholly in agreement with this, but unfortunately due to the very limited study budget we were unable to offer kidney function tests to the participants.  

 "Please provide more details on how you did the radiography. Was the interpretation done centrally or was this as used in routine practice? What happened if there was a disagreement in the 10% of images which were randomly selected for routine control?"

 In the exclusion criteria; was diabetes only on self report? How reliable is this in excluding DM? I believe this would be a great confounder in the size of the kidney as would HIV since the kidneys tend to be bigger. Many DM patients do not know that they have it." 

**Response:** Thank you for raising this important point. The quality control was done externally by two experienced consultant radiologists. After the initial quality check, specific feedback and further training was given to the sonographers on image optimisation. After the final quality check, 4/41 images (10%) were deemed unusable and were excluded from the analysis.   Diabetes was on self-report and so unfortunately yes, there is likely to be unknown undiagnosed diabetes among the cohort. This could potentially have biased kidney sizes to larger dimensions. Given that we found kidneys to be small in size, this potentially makes our findings even more interesting. However, the population prevalence of diabetes in 20-79 year olds in Malawi is 7.3% ^1^.  

 "The standardization for body size is a strong point for this study. Is there any major difference in size between the rural and urban dwellers in the study and overall in Malawi?"  

**Response:** Thank you for raising this most interesting question. We have looked into it and compared height and BMI between the urban and rural locations and found no evidence of a difference (please see the box plots found in the PDF file linked here).
